# What is public health ethics for the geriatric community?

**DOI:** 10.4102/hsag.v27i0.1824

**Published:** 2022-09-27

**Authors:** Laetus O.K. Lategan, Gert J. van Zyl, Willem H. Kruger

**Affiliations:** 1 Division of Research, Innovation and Engagement, Central University of Technology, Bloemfontein, South Africa; 2 Faculty of Health Sciences, University of the Free State, Bloemfontein, South Africa; 3 Department of Community Health, Faculty of Health Sciences, University of the Free State, Bloemfontein, South Africa

**Keywords:** care ethics, geriatric community, professional ethics, ethics, public health

## Abstract

**Background:**

A growing ageing community puts additional demands on the public health system. This will contribute to ethical consequences for the health care sector. A public health ethics framework can contribute towards addressing the ethical challenges faced by the geriatric community.

**Aim:**

This article intends to contribute to a public health ethics framework for the geriatric community from a South African perspective.

**Setting:**

Twenty-two participants from six geriatric institutions, two each in the three provinces, participated in the research. The provinces are the Free State, Northern Cape and North West.

**Methods:**

Fifteen statements were rated using a five-point Likert scale questionnaire. The statements were grouped into three indexes, namely what is ethics, what is public health ethics and what is public health ethics for the geriatric community?

**Results:**

Ethical behaviour is observable not only from person to person but also through systems, processes and practices. The need is to understand *how* to apply ethical principles to the working environment. A public health ethic can be understood from applied, professional and social ethics.

**Conclusion:**

Public health ethics is the application of health care principles through a professional ethic resulting in care and relationship-building. The core of what public health is should be the basis to identify a public health ethic where the focus is on the community and improvement of the quality of health and well-being of the community.

**Contribution:**

No evidence of a public health ethics framework for the geriatric community could be identified in South Africa.

## Introduction

Vulnerability is a growing challenge, especially among geriatric people who are represented in a rising ageing and consequently elderly or geriatric community. Statistics from the World Health Organization (WHO 2015:43) suggested that the world population older than 60 years will nearly double by 2050. World Health Organization identifies this growth as a global phenomenon. The population comprising 65 years and older is in general referred to as the elderly or geriatric community (Dotchin et al. 2013), although the statistical evidence often includes people older than 60 years when geriatric populations are discussed. In Africa, the population 60 years and older is referred to as geriatric (Naidoo & Van Wyk 2019:1).

According to the 2017 statistics in South Africa, 8.1% of the population is older than 60 years (Republic of South Africa [RSA] 2017:3). The 2020 Mid-year Population Estimates Report indicates that the number of people older than 60 years is 9.1% of the population (RSA 2020a:5). This report states that the population of 60 years and above increased by 1.9 million people from 2002 to 2020. This growth represents an increase of 1.1% from the period 2002 to 2003 and 3.0% from the period 2019 to 2020 (RSA 2020a:10, 12). It is estimated that the geriatric population in sub-Saharan Africa will increase from 42.6 million in 2010 to 160 million in 2050 (Naidoo & Van Wyk 2019:1).

A growing elderly or geriatric community will place more demands on the already challenged social and health services. This, in turn, will put more strain on the geriatric community as a vulnerable community. The geriatric community may be vulnerable because of ongoing social factors negatively impacting this community’s health, of which health care provision in general and challenges with the quality of health care provision are important contributing factors. The coronavirus disease 2019 (COVID-19) pandemic, caused by the severe acute respiratory syndrome coronavirus-2 (SARS-CoV-2), is adding to increased economic and health care vulnerability. It was projected with the breakout of the pandemic that 50% of people dying from COVID-19 would be older than 80 years (WHO 2020a, 2020b).

The consequences of a growing geriatric community are well described by the WHO’s references to the demands an ageing population and society will place on health care provision (WHO 2015, 2017). It is generally assumed that these consequences will not be without ethical challenges and therefore require ethical guidelines, as commented by Chan (2017). The growing impact of social determinants on health requires ethical considerations when public health services are identified and implemented. The geriatric community can benefit from a public health ethics framework because of their health, social and financial vulnerability.

Chan’s remark raised the question of whether there is (at least) a guiding definition and therefore scope of what public health ethics for the geriatric community is. Although it will be unfair to claim that there is no sufficient definition or scope of activities, within a South African context such reference is absent as confirmed by publication indexes such as ScienceDirect, ProQuest, Taylor and Francis and Sabinet African Journal Collection. On the official website of the National Department of Health, no such policies could be identified.

This article aims to contribute to the development of a public health ethics framework for the geriatric community. Core to such a framework will be to understand what public health ethics is for the geriatric community.

## Definitions

In dealing with the given facts, the departing point was Beauchamp and Childress’ (2013, first published 1979) emphasis on the respect for *beneficence, nonmaleficence, autonomy* and *justice* as principles for bioethics. These principles, however, are generally regarded in health care as the backbone for ethics in activities related to health and medicine. In a seminal discussion initiated by Gillon (1994), the given principles were extended by adding ‘scope’ to them. This recommendation has since then commonly been known as the ‘four principles plus scope’ approach. Gillon argued that regardless of personal belief, orientation and affiliation, any person could commit to these principles. He referred to the four principles as *prima facie* principles, meaning that they are binding unless in conflict with another moral principle. ‘Scope’ raised the concern of *if* and *how* these principles are applied and what their subsequent consequences could be. The need to add ‘scope’ to these principles can further be informed by Ten Have, Ter Meulen and Van Leeuwen (2013:8–11), who argue that ethics deals with either external factors (e.g. the influence of a group or a community) or internal factors (one’s own attitude or belief system), guiding a person or agency to act in a particular manner. The summative interpretation is that ethics is based on principles.

This general understanding of the nature of ethics invites a general understanding of public health too. Berridge’s discussion of public health influenced the approach taken in this article to phrase a working definition and scope for public health. Although public health exists beyond a single definition, Berridge (2016:2, 69) argued that public health refers to a profession and a body of knowledge. In its narrowest sense, public health refers to the health of a population, longevity of individual members and freedom from disease. Public health also has prevention of illness rather than the provision of health and well-being as an anticipatory character. This approach can be aligned with Baylis, Kenny and Sherwin (2008), who argued for an ethics framework serving the public health needs to prevent illness, build physically and socially healthy communities and eliminate health inequities.

As a working definition for this article, *public health* refers to *the organised strategies, interventions and services to promote the quality of health and well-being of a community or population* (cf. Lategan & Van Zyl 2018 for a detailed discussion on defining public health). This definition also identifies the expectations associated with public health, notably the quality of service and health care provision.

On the basis of the given discussion on public health and ethics, *public health ethics* can be defined as the ethical principles, decisions and behaviour to improve a community or population’s health and well-being.

From this definition, the identified scope of public health ethics is (1) the identification and application (2) of ethical health care principles (3) to secure the quality of health care provision and services for a targeted population (4) to result in the improvement of health and well-being. This understanding of public health resonates with three ‘thought schools’ in ethics, namely principle identification (virtue ethics), action and behaviour (deontology) and the outcome of the applied principles (consequentialism) (Mautner 1997:180–181, 593).

The public health’s focus on the population suggests two differences between public health ethics and medical ethics and bioethics. Where medical ethics and bioethics focus on the individual (e.g. the doctor–patient relationship or death and dying), the focus in public health ethics is on the population or a community. The focus is therefore not on individual needs but rather on the community’s needs. The focus is also on prevention and not therapeutic or clinical intervention.

Three approaches were taken in profiling what public health ethics is for the geriatric community. These were:

1. *applied ethics* as it addresses the application of principles in service delivery and provision
2. *professional ethics* as it calls on the behaviour towards vulnerable groups
3. *social ethics* as it concerns itself with groups.

These approaches were used in the discussion of the research results presented in the Result and Discussion Section.

The approach taken was informed by comments offered by Baylis et al. (2008:4) that the four *prima facie* principles identified for bioethics cannot simply be applied to public health. The bioethical principles deal with the conflict between individuals in a clinical situation. Public health ethics instead ‘must begin with a recognition of the values at the core of public health, not a modification of values used to guide other kinds of healthcare interactions’.

The intended outcome of this article is to identify what constitutes public health ethics for the geriatric community. The outcome of the article is informed by the ethical needs caused by a growing elderly community. The focus on public health is the added value to the lives of the geriatric community.

## Method

A comprehensive literature review on ethics relevant to geriatric care was completed (Lategan 2021). Fifteen statements were identified from the literature review and grouped into three indexes, namely what ethics is, what public health ethics is and what public health ethics is for geriatric people. These statements form part of a broader study to develop a public health ethics framework for the geriatric community. This study has a total of 50 statements covering three indexes, namely social determinants, public health ethics and a public health ethics framework.

A questionnaire was used to sample the data, where statements were rated using a Likert-type scale. *This article will discuss the results of the rating of the statements only*. The rating of the 15 statements on public health ethics contribute towards measuring the participants’ opinions and perceptions of public health ethics. The purpose of this analysis was to identify what constitutes public health ethics for the geriatric community.

For this scale, Cronbach’s alpha was 0.866 and Cronbach’s alpha based on standardised items was 0.905. These results indicated a high level of internal consistency for the 15 items used in the scale.

The statements were rated by means of a five-point Likert scale table where the rating took place according to the ‘least important’ (represented by 1) to ‘cannot do without this’ (represented by 5). The rating of statements can fit the two extremes of the Likert scale, namely ‘agree’ or ‘disagree’, with a moderate or neutral point being indecisive.

The use of a five-point Likert scale was based on respondents having choices without losing focus because of too many possibilities to choose from. This consideration was informed by the aim of the study to develop a public health ethics framework for the geriatric community and the profile of participants, namely doctors, registered nurses, nurses, health care workers, managers and administrators who all may not have prior experience to participate in questionnaires.

The data were sampled through completing questionnaires by 22 participants from six geriatric institutions who agreed to participate in completing the questionnaire. This took place from July 2020 to August 2020. As a result of COVID-19 regulations from March 2020 to September 2020, it was not permissible for the researcher to visit the institutions. The sampling of data took place during the time of national lockdown restrictions Level 3 (from June 2020 to 16 August 2020) and Level 2 from 17 August 2020 to 20 September 2020. The lockdown restrictions fell under the national state of disaster announced on 15 March 2020.

Because of these regulations, the researcher called the manager at each of these institutions telephonically and requested permission to engage with staff at the institution. Based on the provision of possible names, the researcher engaged them individually on the scope of the research with at least written background information and at a request for their participation and consent. Hence, no pilot study was carried out. The questionnaires were sent via courier services separately in sealed envelopes addressed to each of the participants. Feedback based on the drafted framework was provided during a webinar for the participants in the study only. The main objective was to give feedback and not to obtain consensus on the outcome of the framework or parts thereof.

The Statistical Package for the Social Sciences (SPSS) statistical support software was used to analyse the data sampled from the Likert scale.

### Ethical considerations

Approval to conduct the study was received from the Health Sciences Research Committee (HSREC), Faculty of Health Sciences, University of the Free State, Bloemfontein (reference number: UFS-HSD2019/0471/2502) and approval to conduct the research was obtained from the managers of the various institutions. Informed consent was obtained from all participants before the start of the data collection. The data were sampled from July 2020 to August 2020 and analysed from October 2020 to December 2020.

### Setting

Six geriatric institutions were identified, two each in the Free State, Northern Cape and North West provinces. These provinces have the smallest populations compared with the other six South African provinces and represent 29.14% of the population older than 60 years (RSA 2020a, 2020b). Economically, these provinces fall outside the mainstream gross domestic product for provinces in South Africa (RSA 2018).

The geriatric institutions and the participants were identified based on Marshall’s (1996:524) grouping of convenience sampling (most accessible environment). Purposeful sampling was also used to identify and select geriatric institutions that are in marginalised provinces and often under-serviced regions and that may not always be part of data collection on a particular topic because of their locality (Palinkas et al. 2015:535).

Twenty-five participants were invited to participate in the study. The invitation was based on the number of participants who voluntarily indicated their willingness to participate in the study. Twenty-two participants from the six geriatric institutions eventually participated in the rating of the statements. The geriatric population was excluded from the data sampling as the focus was on the gathering of in-depth information on public health ethics as perceived by the identified target population, that is, the health care providers and managers. The profile of the participants confirmed two groups, one with medical or health care experience (49.9% of respondents) and one with management or administrative experience (45.4% of respondents). The information confirmed a high percentage of post-school training (72.7% of respondents), with 36.4% respondents having cumulatively 21 years and more of work experience. The different job profiles and therefore work responsibilities of the participants and diversity in location based on geographic area (province and city or rural) secured the multiplicity in participation.

## Results and discussion based on the rating of statements

The analysis of the data presented the following results.

Firstly, there is an 88.18% percentage of agreement with statements on what ethics is and its meaningful contribution towards dealing with the geriatric community (Statements 1 to 5 & 8 to 15; Table 1). The calculation of the interquartile range and standard deviation is not applicable in the case of categorial data.

**TABLE 1:** Rating of statements around public health ethics.

Statements around public health ethics	Strongly disagree (%)	Disagree (%)	Neutral (%)	Agree (%)	Strongly agree (%)
1. Ethics can be explained as the choice between what is good and what is bad.	0.0	0.0	4.5	68.2	27.3
2. Ethics is about having the best interest of a person and situation at heart.	0.0	0.0	0.0	65.0	35.0
3. Ethics is not about the needs of the other only but also the self.	0.0	4.8	9.5	61.9	23.8
4. Ethics is about dealing with the vulnerability of the self, other people, systems and the immediate situation.	0.0	0.0	4.8	57.1	38.1
5. Ethics is knowing what one needs to do right to prevent harm to the self, other people, systems and the immediate situation.	0.0	0.0	4.5	54.5	40.9†
6. Ethics is influenced by one’s own understanding of what is good for the self, other people, systems or a situation.	0.0	4.5	0.0	63.6	31.8†
7. Ethics is influenced by one’s liking or disliking of other people or systems.	13.6	13.6	18.2	40.9†	13.6
8. Ethics is not only about people but also about systems, practices, processes and application.	0.0	0.0	4.5	63.6	31.8†
9. Public health ethics for geriatric people is about what is best to promote the health of this population group.	4.5	0.0	4.5	54.5†	36.4
10. There are not enough ethical guidelines available to support healthy living conditions for geriatric people.	0.0	4.5	18.2	59.1†	18.2
11. Public health ethics can play a role in the prevention of poor health.	0.0	0.0	0.0	63.6	36.4
12. Public health ethics for geriatric people is about care and relationship-building between various stakeholders and geriatric people.	0.0	0.0	22.7	50.0	27.3
13. Public health ethics for geriatric people should make health care practitioners more sensitive towards the vulnerability of geriatric people.	0.0	0.0	4.5	59.1	36.4
14. Public health ethics for geriatric people deals with the fairness of how geriatric programmes are implemented.	0.0	0.0	9.1	86.4	4.5
15. Public health ethics for geriatric people should change behaviour towards elderly communities.	0.0	0.0	9.1	72.7	18.2

*Source*: See Lategan, L.O.K., 2021, ‘A public health ethics framework for the geriatric community: A South African perspective’, PhD thesis in Community Health, University of the Free State, Bloemfontein

† , The percentages are rounded up to first decimal place.

The high percentage of agreement confirmed the generally accepted interpretation of what ethics is, that the geriatric community’s vulnerability should be a focus in public health ethics and that ethics should address harm (whether caused through people, structures, systems or processes). That there is an accepted understanding of what ethics is (or in this case, what public health ethics is) could be a contributing factor to the little attention given to defining public health ethics in literature. This raised the question of whether the real matter is to understand what public health ethics is. Should the debate not have a different focus? When the doctors, health care practitioners, workers, managers and administrative officials consider the application of principles in their working environment, should they not have knowledge thereof? Therefore, there is less need of defining what public health ethics is and a greater need for understanding what the task of public health ethics is. Statement 10 contributed to this remark, with 77.3% of respondents who agreed or strongly agreed that there are not enough guidelines available to support healthy living conditions for the geriatric community. In return, the rating of this statement linked with the need for a better understanding of deontology and consequentialism.

The question was raised as to whether a professional or an institutional code is merely perceived to be what one must do to stay out of trouble, rather than using the principles to direct decisions on what must be performed in the workplace. Verbruggen (2013:161–162) commented that professional ethics is more than personal morality. Professional ethics is a justifiable public intervention in people’s lives – in this case, their health. She further commented that professional ethics is not about the application of universal ethical principles but rather a professional engagement with people in a particular context based on these principles. Grypdonck, Vanlaere and Timmerman (2018) argued that the focus should not be on one’s own agenda but on what the essential need of the receiver is. The high rating (agree or strongly agree) of Statements 13 to 15 suggests that there should be more sensitivity towards the vulnerability of the geriatric community (95.5%), fairness in implementation of programmes (90.9%) and a changed behaviour towards the geriatric community (90.9%).

Secondly, the individual’s orientation towards the role of the self or personal likes or dislikes invited mixed responses. Statement 7 confirmed the agreement or strong agreement that a personal view influences ethics (54.3%). The same rating represented 27.2% of respondents, who disagreed or strongly disagreed with the statement. The significance of this observation confirmed the role of the individual in ethics, more so in a professional context that is often challenged by work skills and experience and cultural or ideological orientation. Several studies confirming that cultural or ideological orientations play no role can be countered by an equal number of studies that will make the opposite case. Instead of pursuing this debate, however, another route is taken, supported by discourse ethics. In the context of discourse ethics, the focus is away from *power* to *reasonable*. Power represents an ideology, while reasonable supposes a consensus on what is doable and achievable. Raymakers (2016) roughly drafted, after Habermas’ Discussion Ethics, a discussion ethic free of domination during the discussion. Statement 11 contributes to the context of this discussion. The 77.3% agree or strongly agree rating contributed to the awareness that care and relationship-building are important contributors to public health ethics. This can be confirmed by Baylis et al. (2008:6), who proposed relational personhood and autonomy as the core for public health and a public health ethic. Statements 4 to 11 further confirm the vulnerability of the geriatric community as an ethical challenge and the positive contribution public health ethics can make towards the health and well-being of the geriatric community. The overall conclusion is that although personal views on ethics cannot be ignored, personal views can be directed to avoid domination in defining or applying ethics. In public health ethics, the focus should be directed away from the individual towards the community, and within the community, care and relationship-building should be core in identifying and applying principles relevant to public health ethics.

Thirdly, the meaning that ethics should have for the individual is a personalistic view and confirms the value of all humans in ethics. This view should not be confused with the interpretation that a person is the supreme value for ethics. This confirmation is consistent with the *prima facie* principles identified in the introduction of this article. Statements 1 to 3 shared a 90% agreement or strong agreement with the statement. Regarding Statement 2, there was 100% certainty that ethics is about what is best for the person or situation. The neutral rating of Statement 3 (9.5%) and the disagreement (4.8%) can be interpreted against the background of personalism, where the self is the absolute doctrine for ethics. The majority of ratings for this statement rejected personalism. The value of this rating is the awareness that ethics is about the other *and* the self, hence not one party only. Statement 8 contributed to a broader view than people as the only focus in public health. The 95.5% agree or strongly agree rating of this statement motivates the perspective that within public health ethics systems, practices and processes cannot be ignored. The statements involved individual responsibility towards ethics. Notable is the high percentage of agree or strongly agree expressed through the rating of statements in favour of actions such as ‘choice’, ‘interest at heart’, ‘dealing’, ‘prevent’, ‘understanding’ and more.

Fourthly, people together with systems, practices, processes and applications are part of the scope of public health ethics, as rated by Statement 8. The 95.4% agree or strongly agree rating endorsed the understanding that ethical systems, practices, processes and applications are important for the health and well-being of the geriatric community. Although this interpretation can be taken as given, similar to what (public health) ethics is, the question arises as to whether public health ethics exists as awareness or whether it is practised as part of health care provision and policy. This question is justifiable, especially in the context of the locations of participating institutions. The participants work in economically and socially marginalised or under-serviced geographical areas. The official websites of the provincial health departments carry no communications on ethics in general, apart from a brief reference to the *Batho Pele* (People First) Principles that commit to quality of service and delivery. (The comment is based on the website information retrieved on 27 March 2021.) Although the *Batho Pele* Principles are not specifically mentioned in the National Department of Health’s Strategic Plan, 2020–2025 (RSA 2020c), this plan does identify the attention that should be given to systems, practices and processes. The summative conclusion from this rating is the agreement that in public health ethics, systems, practices and processes cannot be ignored. The mere assumption that systems, practices and processes are ethical calls for managing this assumption through assessing compliance with professional codes. Crane and Matten (2004:151) advised that within these codes there should be general and specific statements. Building on their advice, a general statement is to respect elderly people and a specific statement is not to take a bribe in return for a service delivered.

Fifthly, the rating presented important interpretations. Only 20% of the statements have no neutral rating (Statements 2, 6 & 11). These statements reflected on what ethics is, the individual’s own understanding of ethics and the positive role ethics can play in preventing poor health. Only Statement 6 had a disagreement rating of 4.5%. From these statements, the conclusion may be drawn that there is a shared understanding of what ethics is and what its value is. These statements covered the nature of ethics, the influence of personal likes and dislikes in ethics and personal engagement with other people. This confirmed the indisputable role that the individual’s own orientation plays towards ethics.

The neutrality rating ranged from 4.5% to 22.7% (Statements 1, 5, 7, 8, 9 and 13). Of note is the 22.7% neutrality view in Statement 12 that public health ethics is about care and relationship-building between various stakeholders and the geriatric community. This observation is further informed by a 0% disagree or strongly disagree rating of the same statement. Three interpretations are viable. Firstly, ethics is understood as deontology – the duties based on the choice between what is right or wrong. Secondly, if read with the 77.3% ‘agree’ or ‘strongly’ agree rating, then the care and relationship-building focus of ethics is further supported. Such an interpretation is more likely, as there was a 0% disagree or strongly disagree rating of the statement. The 94.4% rating in Statement 8 can further contribute to this interpretation. This interpretation confirms a trend in the rating of statements, namely that deontology is important in public health ethics. Another observation is that only three statements have a disagree rating ranging between 4.5% and 4.8% (Statements 3, 6 and 10), which have limited influence on the interpretation of the rating. Two of the statements (3 and 6) referred to the role of the individual in determining what ethics is. Statement 10 dealt with the availability of guidelines. The disagree rating of 4.5% has no significant meaning on its own. Even if it is read with the neutrality rating of 18.2%, it does not influence the positive rating of this statement. Statement 9 has a strong disagreement rating of 4.5%, which most probably relates to an understanding of what public health ethics is. Statement 7 is the only statement with ratings in all five categories. This underlined the role of the individual’s liking or disliking of other people or systems.

Lastly, these ratings must be understood against the almost 50–50 representation from the (1) health care practitioners and workers and (2) management and administration cohorts who responded to these statements. This serves in general as agreement on what ethics and public health ethics are and what the identified role is for public health ethics.

The results of the given data collection presented here not only delineate the space of public health ethics as *applied ethics* but also create the link to *professional ethics*. The results further identified the need for a *care ethics* as social ethics.

Baylis et al. (2008) argued that the known principles for bioethics cannot simply be changed to fit public health ethics. What public health is cannot be ignored and should be the point of departure. The core focus of public health is the community or population and not the individual. This does not mean that the individual has no responsibility or that all responsibilities are delegated to the group. Virtue ethics applies to the moral character of the individual and the group. The reference of Baylis et al. (2008:10) to relational responsibility, involves the individual and the group. The binding factors are a recognition of mutual vulnerability and interdependency. Gillon’s four principles plus scope contribute to the awareness that both the individual and the group’s orientation towards ethics influences the application of ethical principles. New discourses in deontology steer away from a stark rationalistic approach, making use of abstract principles for ethics practices to a more hermeneutical approach and focus on practices, stories and codes. The revival of deontology emerged from a growing interest in applied ethics (such as professional ethics) (Raymakers 2016:53). Care ethics contributes meaningfully to acknowledging power plays in dealing with ethical challenges in practice and focuses on relationship-building when dealing with these challenges (Tjong Tjin Tai 2014:196–197).

From these results and discussions, the following refined definition for public health ethics may be offered: *public health ethics is the application of ethical principles through a professional ethic resulting in care and relationship-building.*

Applied to the geriatric community, the community’s vulnerability will guide the application of the principles. Professional ethics’ and care ethics’ specific contribution will be to avoid power domination and protect vulnerability.

### Limitations

This article represents a part of a comprehensive study on a public health ethics for the geriatric community. As a result of the specific focus of the article, the different nuances from the study cannot be recorded. This article focuses only on the rating of statements sampled through a questionnaire on what public health ethics is. This study was limited to health care providers and managers only and excluded the geriatric population. The participants presented three marginalised provinces. The conclusions based on the analysis of sampled data are limited to those who participated in the study.

## Conclusion

In this article, 15 statements were identified to help define public health ethics. From the rating of the statements, it was confirmed that promotion of health and well-being through the quality of service and health care provision are the *foci* of public health. The health and well-being of geriatric people are challenged through vulnerability and harm caused by people, institutions, systems, practices, processes and application. This is evident through the availability and the quality of service and health care provision.

The rating of statements contributes to a less abstract understanding of public health ethics, namely principle identification. The statements created a broader interpretation of public health ethics, such as the requirement of professional behaviour and addressing the vulnerability of the geriatric community through care and relationship-building. It is also apparent that public health ethics calls on various stakeholders to secure ethical behaviour when dealing with the geriatric community. The application of public health ethics can best be delivered through professional ethics and care ethics.

Applied to the geriatric community, the application of principles, decisions, professional behaviour and care will consider the specific health care needs of this community.

From these observations, the following can be confirmed:

- Public health is about the community or population, the attainment of health and prevention of disease and improving quality of services in support of health and well-being of the group.
- Individual responsibility can never be removed from the community.
- Although bioethical principles such as respect for beneficence, nonmaleficence, autonomy and justice were initially identified to deal with individual challenges around life and death, these principles have value for public health ethics as the principles subscribe to the basis of all ethics: *do no harm*.
- The rating of statements confirms that although it is good to have a shared understanding of what ethics or public ethics is, the need is more for applying ethical principles in pursuing the objectives of public health.
- Professional ethics supported by a care ethics approach is beneficial for applying public health principles.
